# Waist‐to‐Body Mass Index Ratio and Risk of Musculoskeletal Disease in Type 2 Diabetes: A Cohort and PhIP‐Seq Analysis

**DOI:** 10.1002/jcsm.70336

**Published:** 2026-08-07

**Authors:** Yongze Zhang, Luxi Lin, Lifeng Zheng, Xiaohuang Lin, Zhaowei Xu, Ximei Shen, Lingning Huang, Fengying Zhao, Sunjie Yan

**Affiliations:** ^1^ Department of Endocrinology, the First Affiliated Hospital Fujian Medical University Fuzhou China; ^2^ Department of Endocrinology, National Regional Medical Center, Binhai Campus of the First Affiliated Hospital Fujian Medical University Fuzhou China; ^3^ Clinical Research Center for Metabolic Diseases of Fujian Province, the First Affiliated Hospital Fujian Medical University Fuzhou China; ^4^ Fujian Key Laboratory of Glycolipid and Bone Mineral Metabolism, the First Affiliated Hospital Fujian Medical University Fuzhou China; ^5^ Diabetes Research Institute of Fujian Province, the First Affiliated Hospital Fujian Medical University Fuzhou China; ^6^ Metabolic Diseases Research Institute, the First Affiliated Hospital Fujian Medical University Fuzhou China; ^7^ Orthopedics Department the First Affiliated Hospital of Fujian Medical University Fuzhou Fujian China; ^8^ Key Laboratory of Gastrointestinal Cancer (Fujian Medical University) Ministry of Education Fuzhou China

**Keywords:** osteoporosis, sarcopenia, type 2 diabetes, waist circumference, waist‐to‐BMI ratio

## Abstract

**Background:**

Musculoskeletal complications in type 2 diabetes (T2DM) are inadequately captured by body mass index (BMI). Waist‐to‐BMI ratio (WBR) may better reflect adverse body composition. We examined cross‐sectional and longitudinal associations between WBR and musculoskeletal disorders in T2DM.

**Methods:**

This two‐phase study was conducted within an ongoing hospital‐based cohort at the First Affiliated Hospital of Fujian Medical University (Fuzhou, China). The cross‐sectional analysis included 4157 adults with T2DM recruited between March 2012 and August 2023 (54.3% men; mean age 59.4 ± 10.3 years), using data from their first assessment. Associations of waist circumference (WC), waist‐to‐height ratio (WHtR), waist‐to‐hip ratio (WHR), BMI and WBR with osteopenia, sarcopenia, sarcopenic osteopenia (SOs), sarcopenic obesity (SOb) and fractures were evaluated. The prospective cohort comprised a longitudinal subset enrolled between March 2012 and June 2022, ensuring at least 1 year of follow‐up prior to administrative censoring in August 2023. A total of 440 individuals (57.0% men; mean age 59.7 ± 9.7 years) were followed for a median of 34.0 months (20.0–57.0). Associations between time‐dependent WBR and incident outcomes were assessed using Cox models. A nested exploratory analysis was conducted within the cohort. Thirty participants with extreme annualised WBR change (ΔWBR/yr) were selected. Baseline serum samples collected at enrolment, prior to outcome occurrence, were analysed using phage immunoprecipitation sequencing (PhIP‐Seq).

**Results:**

Cross‐sectionally, WBR was negatively correlated with bone mineral density and appendicular skeletal muscle mass index and positively correlated with osteopenia, sarcopenia, SOs, SOb and fractures (all *p* < 0.01), whereas BMI, WC, WHtR and WHR showed weaker associations. After adjustment, higher WBR was independently associated with osteopenia (men: OR 1.723, 95% CI 1.614–1.840; women: OR 1.420, 1.348–1.495), sarcopenia (men: OR 4.779, 4.165–5.484; women: OR 2.991, 2.683–3.334), SOs (men: OR 6.261, 5.314–7.377; women: OR 4.336, 3.753–5.010), SOb (men: OR 4.737, 3.975–5.646; women: OR 4.652, 3.715–5.825) and fractures (men: OR 1.236, 1.093–1.397; women: OR 1.103, 1.003–1.213; all *p* < 0.05). Prospectively, higher time‐dependent WBR predicted incident osteopenia (HR 1.365, 95% CI 1.024–1.820), sarcopenia (HR 1.282, 1.086–1.512), SOs (HR 1.408, 1.176–1.686), SOb (HR 1.634, 1.262–2.116) and fractures (HR 1.369, 1.029–1.821). PhIP‐Seq analysis identified differential autoantibody reactivity related to muscle structural organisation and cytoskeletal regulation, while bone‐related differences were enriched in Wnt signalling and hormone‐related pathways.

**Conclusions:**

Higher WBR and longitudinal increases were independently associated with osteopenia, sarcopenia, sarcopenic phenotypes and fractures in individuals with T2DM.

**Abbreviations**
AGE: advanced glycation end‐product
AKT: protein kinase B
ALB: albumin
ALT: alanine aminotransferase
ASM: appendicular skeletal muscle mass
ASMI: appendicular skeletal muscle mass index
AST: aspartate aminotransferase
AUC: area under the curve
BF: body fat percentage
BMD: bone mineral density
BMP: bone morphogenetic protein
BMI: body mass index
Ca: calcium
CI: confidence interval
CKD: chronic kidney disease
CT: computed tomography
DKD: diabetic kidney disease
DR: diabetic retinopathy
DPN: diabetic peripheral neuropathy
DXA: dual‐energy X‐ray absorptiometry
eGFR: estimated glomerular filtration rate
EPV: events per variable
FC: fold change
FRAX: Fracture Risk Assessment Tool
GO: Gene Ontology
HbA1c: glycated haemoglobin
HC: hip circumference
HDL‐C: high‐density lipoprotein cholesterol
HF: hip fracture probability
HR: hazard ratio
IQR: interquartile range
KEGG: Kyoto Encyclopedia of Genes and Genomes
LDL‐C: low‐density lipoprotein cholesterol
MOF: major osteoporotic fracture probability
NF‐κB: nuclear factor kappa B
OR: odds ratio
PI3K: phosphoinositide 3‐kinase
PhIP‐Seq: phage immunoprecipitation sequencing
ROC: receiver operating characteristic
SGLT2i: sodium–glucose cotransporter‐2 inhibitors
SOb: sarcopenic obesity
SOs: sarcopenic osteopenia
T2DM: type 2 diabetes mellitus
TD‐Cox: time‐dependent Cox regression
UACR: urinary albumin‐to‐creatinine ratio
VAT: visceral adipose tissue
VEGF: vascular endothelial growth factor
WHR: waist‐to‐hip ratio
WHtR: waist‐to‐height ratio
WBR: waist‐to‐BMI ratio
WC: waist circumference

## 1 Introduction

The global obesity epidemic increases the risk of cardiovascular disease, type 2 diabetes mellitus (T2DM), certain cancers and all‐cause mortality [1]. Despite its widespread use, body mass index (BMI) cannot distinguish between adipose and lean mass, failing to accurately reflect body composition, a limitation that clinically manifests as the ‘obesity paradox’ [2, 3]. This phenomenon describes the counterintuitive association between higher BMI and improved survival in certain chronic conditions, including T2DM, indicating that BMI alone may underestimate the health implications of adverse body composition.

This limitation is especially consequential in T2DM, a systemic metabolic disease marked by multi‐organ dysfunction and elevated mortality [4]. Despite advances in understanding microvascular and macrovascular complications, musculoskeletal involvement in T2DM remains poorly understood. Sarcopenia, characterised by progressive loss of skeletal muscle mass and function, is associated with markedly elevated fall, disability and mortality risks [5]. When coexisting with obesity, sarcopenia gives rise to sarcopenic obesity (SOb), a high‐risk phenotype associated with exacerbated metabolic dysfunction, chronic inflammation and insulin resistance [6]. Patients with T2DM exhibit elevated risks of osteoporosis [7] and fragility fractures [8]. Although traditional perspectives have suggested a protective effect of obesity on skeletal health through increased mechanical loading and adipose‐derived oestrogen production [9], emerging evidence indicates a heightened risk of major osteoporotic fractures among individuals with obesity [10].

Given that obesity affects approximately 44% of patients with diabetes [11] and sarcopenia (31.1%) [12] and osteoporosis are highly prevalent among individuals with T2DM, there is an urgent need for more refined assessment tools. Alternative waist circumference (WC)–derived indices, including waist‐to‐height ratio (WHtR) and waist‐to‐hip ratio (WHR), have been proposed to overcome the limitations of BMI by more accurately reflecting visceral adiposity. These metrics correlate with sarcopenia risk [13] and fracture incidence [14], demonstrating improved sensitivity to body composition changes. Their ability to comprehensively and simultaneously predict the incidence and progression of the full spectrum of musculoskeletal disorders, including osteopenia, sarcopenia and their comorbid phenotypes, remains limited.

This study evaluates WC and its derived indices, including WHtR, WHR and the novel waist‐to‐BMI ratio (WBR), in relation to musculoskeletal disorders in individuals with T2DM. We hypothesised that WBR, by integrating central adiposity with overall body mass, would improve the identification of individuals at higher risk of musculoskeletal disorders compared with conventional anthropometric indices, thereby supporting risk stratification in this vulnerable population.

## 2 Methods

### 2.1 Study Design and Setting

**FIGURE 1:**
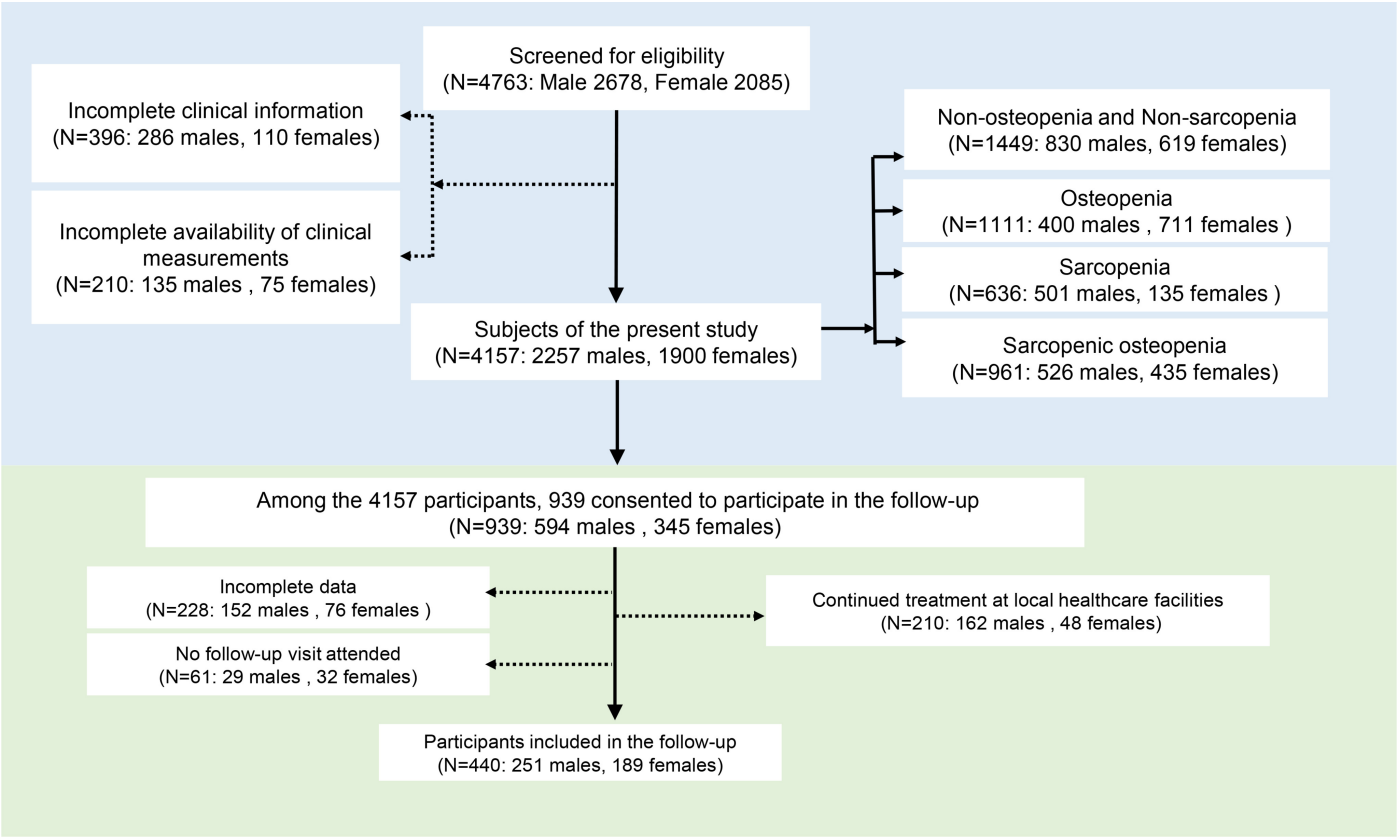
Flowchart of participant selection.

This two‐phase study comprised a cross‐sectional analysis followed by a prospective cohort study. The cross‐sectional phase was conducted to evaluate the associations of WC‐derived anthropometric parameters, including WC, WHtR, WHR and WBR, with musculoskeletal outcomes (osteopenia, sarcopenia, fractures, SOb and sarcopenic osteopenia [SOs]), to identify the anthropometric indicator showing the strongest association. The prospective cohort phase focused on this selected parameter to examine whether its longitudinal changes were associated with disease progression (Figure 1).

Between March 2012 and August 2023, adults with T2DM were continuously and prospectively enrolled in an open cohort at the First Affiliated Hospital of Fujian Medical University, a tertiary teaching hospital in Fujian Province, China, and its Clinical Research Center for Metabolic Diseases of Fujian Province. Participants entered the cohort on different calendar dates and were followed thereafter according to a standardised protocol.

For the cross‐sectional analysis, data from each participant's first assessment after enrolment were used. The prospective cohort comprised a longitudinal subset of this population. Participants enrolled between March 2012 and June 2022 were included to ensure at least 1 year of follow‐up before administrative censoring in August 2023. At least two follow‐up assessments were required.

**FIGURE 2:**
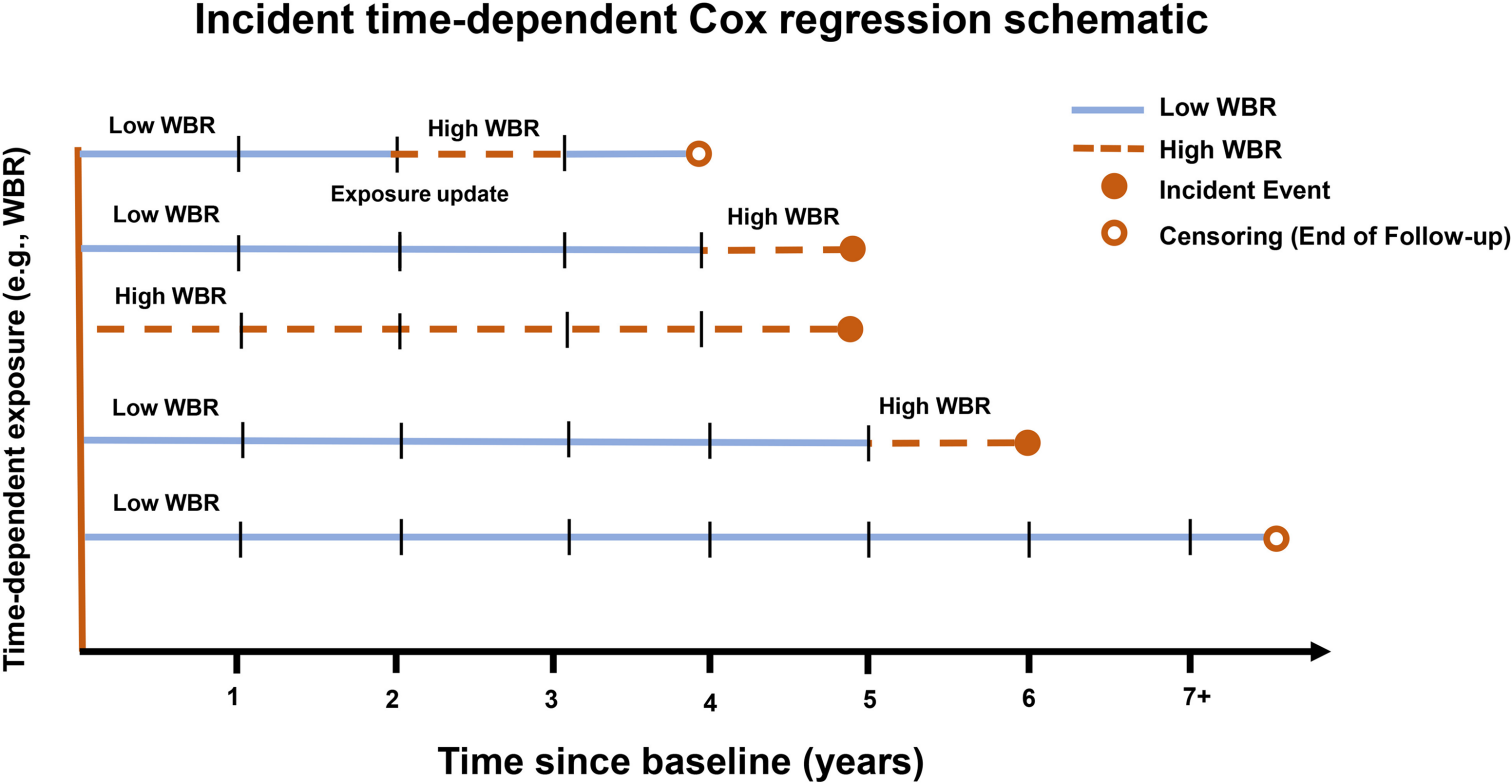
Schematic illustration of the incident time‐dependent Cox regression model. Each horizontal line represents follow‐up time for an individual participant from baseline. The exposure of interest (e.g., WBR) was treated as a time‐dependent variable and updated at each follow‐up visit (black tick marks). Participants could transition between exposure categories (low WBR, solid blue line; high WBR, dashed orange line) during follow‐up. Incident events are indicated by filled circles, and censoring at the end of follow‐up is indicated by open circles. Hazard ratios were estimated using Cox proportional hazards models with time‐dependent exposures, with person‐time allocated according to the updated exposure status at each interval.

Follow‐up visits were scheduled quarterly for routine assessments of glycaemic control and medication adherence, and annual evaluations were conducted for surveillance of diabetes‐related complications, including neurological examinations, biochemical profiling and dual‐energy X‐ray absorptiometry (DXA) (Figure 2).

The cohort was originally established with approval from the Ethics Committee of the First Affiliated Hospital of Fujian Medical University (Approval Nos. 2011.04 and 2012.11). Longitudinal follow‐up and extended biomarker analyses, including phage immunoprecipitation sequencing (PhIP‐Seq), were conducted under an updated protocol approved by the same Ethics Committee (Approval No. 2017.131). Written informed consent was obtained from all participants.

### 2.2 Participants

Adults with T2DM diagnosed according to the 1999 World Health Organisation (WHO) criteria [15] were eligible for inclusion. The exclusion criteria were other types of diabetes (type 1, gestational or secondary diabetes); acute or critical illness, including diabetic ketoacidosis or hyperosmolar coma; and comorbid conditions known to substantially affect muscle or bone metabolism, such as genetic disorders, severe malnutrition, hyperthyroidism or cerebrovascular disease.

### 2.3 Sampling Strategy and Matching

Considering the epidemiological characteristics of osteoporosis in T2DM, along with the lower incidence of osteoporosis and higher prevalence of osteopenia in men, stratified sampling was adopted in the cross‐sectional phase to optimise the sample size while maintaining methodological rigour. The participants were stratified by sex and categorised into four clinical phenotypes: non‐osteopenia/non‐sarcopenia, osteopenia alone, sarcopenia alone and concurrent SOs. After excluding participants with incomplete clinical data, age‐based matching was applied under predefined age distribution constraints to ensure comparability across phenotypic groups. The smallest phenotypic subgroups (men with osteopenia alone and women with sarcopenia alone) were used to define the reference age distributions. Participants in the other phenotypic groups were retained if their age fell within the corresponding reference ranges, resulting in variable matching ratios (approximately 1:1 to 1:6) to maximise case retention. Individuals whose age fell outside the predefined age range were excluded from the final analytical sample. The participant selection and matching process is summarised in Figure 1.

### 2.4 Data Collection and Anthropometric Measurements—Exposure

Comprehensive clinical data were collected using standardised case report forms, including demographic characteristics (age and sex), diabetes duration, chronic complications, medical history, family history, smoking status, alcohol consumption and medications.

Anthropometric measurements were performed by trained staff according to standardised protocols. All measurements were obtained in the morning after overnight fasting, with participants wearing light clothing and no shoes. Body weight and height were measured using a calibrated scale (RGZ‐120‐RT, Jianmin, China), and BMI was calculated as weight (kg) divided by height squared (m^2^). WC was measured at the midpoint between the lower rib margin and the iliac crest, and hip circumference (HC) at the level of the maximal gluteal protrusion, using a non‐stretchable tape. All measurements were recorded to the nearest 0.1 cm. WHtR, WHR and WBR were calculated as WC/height, WC/HC and WC/BMI, respectively. Bone mineral density (BMD) and anthropometric measurements were obtained at baseline and during annual follow‐up. Additional assessments were conducted at the time of incident fracture, when applicable. Given variability in the follow‐up duration and outcome timing, longitudinal changes in anthropometric indices were expressed as annualised rates, defined as the difference between baseline and the assessment at outcome ascertainment or, if censored, the last available assessment prior to censoring, divided by follow‐up time (years).

### 2.5 Laboratory Analyses and Body Composition Assessment

Venous blood samples were collected after overnight fasting (≥ 8 h). Laboratory measurements included serum albumin, liver enzymes (alanine aminotransferase, aspartate aminotransferase), serum creatinine, lipid profile (total cholesterol, triglycerides, high‐density and low‐density lipoprotein cholesterol), fasting plasma glucose and glycated haemoglobin (measured by high‐performance liquid chromatography). Estimated glomerular filtration rate (eGFR) was calculated using the Modification of Diet in Renal Disease equation adjusted for sex. Morning spot urine samples were analysed for urinary albumin and creatinine concentrations, and urinary albumin‐to‐creatinine ratio (UACR) was calculated. Body composition and bone parameters were assessed by DXA (Lunar Prodigy, GE Lunar, United States). Appendicular skeletal muscle mass (ASM), total fat mass, BMD and T‐scores were obtained at the lumbar spine (L1–L4), left femoral neck and left total hip. The ASM index (ASMI) was calculated as ASM/height^2^, and body fat percentage (BF) was calculated as total fat mass divided by body weight. Fracture risk was estimated using the FRAX tool, incorporating age, sex, anthropometric variables, prior fracture history, parental hip fracture, smoking status, glucocorticoid use, rheumatoid arthritis and secondary causes of osteoporosis, to estimate the 10‐year probabilities of major osteoporotic and hip fractures.

### 2.6 Diagnostic Criteria

#### 2.6.1 Diabetic Chronic Complications

Diabetic retinopathy (DR) was diagnosed by specialist ophthalmologic assessment using standardised dilated fundus examination. Diagnosis required the presence of characteristic retinal lesions, including microaneurysms, retinal haemorrhages or hard exudates, consistent with established diagnostic criteria [16]. DR severity grading was not further categorised, and the presence of any DR was considered for analysis. Diabetic kidney disease was defined as either persistent eGFR < 60 mL/min/1.73 m^2^ or UACR ≥ 30 mg/g, documented on at least two occasions over a minimum duration of 3 months, after exclusion of non‐diabetic renal pathologies [17]. Diabetic peripheral neuropathy was diagnosed based on standard clinical criteria after excluding alternative aetiologies, such as toxic or hereditary neuropathies. In symptomatic individuals, the presence of abnormalities in one or more of the following neurological examinations supported the diagnosis: pinprick sensation, 10‐g monofilament testing, temperature perception, 128‐Hz tuning fork vibration sense or ankle reflexes. In asymptomatic individuals, at least two abnormal test results were required to establish the diagnosis [18].

#### 2.6.2 Bone Health Definitions‐Outcome

According to WHO standards, osteopenia was defined as a DXA‐measured BMD T‐score < −1.0 at central skeletal sites (L1–L4, femoral neck, or hip) [19]. Although these diagnostic thresholds were originally established for postmenopausal women and men aged ≥ 50 years, they were applied to all participants for consistency in comparative analyses. Fractures were documented based on clinical history (trauma, falls, localised deformity or functional impairment) with radiological confirmation by X‐ray or computed tomography (CT).

#### 2.6.3 Definitions of Sarcopenia and Related Conditions—Outcome

Sarcopenia was defined according to the International Working Group on Sarcopenia criteria as low ASMI, measured by DXA and normalised to height^2^, with cutoff values < 7.23 kg/m^2^ for men and < 5.67 kg/m^2^ for women [20]. SOb was diagnosed in individuals who met the criteria for sarcopenia and had concurrent elevated BF (> 25% for men, > 35% for women), as assessed by DXA. The composite condition of SOs was operationally defined as the coexistence of sarcopenia and osteopenia in the same individual.

### 2.7 Antibody Repertoire Profiling and Bioinformatic Analysis by PhIP‐Seq

To explore immunological signatures associated with dynamic changes in body fat distribution, antibody profiling using PhIP‐Seq was performed in a subset of participants from the prospective cohort. From the 440 individuals included in the longitudinal analysis, participants were ranked according to their annualised change in WBR (ΔWBR/yr), calculated as the difference between baseline and final follow‐up WBR divided by follow‐up duration. A purposive extreme‐phenotype sampling strategy was applied to maximise biological contrast and discovery power. Specifically, 17 participants from the upper tail of the ΔWBR/yr distribution (largest increases) and 13 participants from the lower tail (largest decreases) were selected for the PhIP‐Seq analysis. All selected participants were free of musculoskeletal outcome events at baseline.

The two groups were comparable in terms of age, sex distribution, diabetes duration and baseline BMI (all *p* > 0.05). Serum samples used for PhIP‐Seq were collected at baseline, prior to the occurrence of the study outcomes, and stored at −80°C until analysis. Given the exploratory nature and limited sample size, the PhIP‐Seq analysis was intended for hypothesis generation rather than causal inference, and the findings may not be directly generalisable to the full cohort.

Serum immunoglobulins were incubated with an M13 phage‐displayed 12‐mer peptide library (New England Biolabs), and antibody‐bound phages were immunoprecipitated with protein G magnetic beads (Thermo Fisher Scientific). Following immunoprecipitation, phage DNA was amplified via a two‐step PCR protocol and sequenced on an Illumina HiSeq platform using 2 × 150‐bp paired‐end reads. The PhIP‐Seq protocol was performed as previously described [21].

Raw sequencing data underwent quality control and normalisation. Differentially enriched peptides were identified using the Wilcoxon rank‐sum test (*p* < 0.05, fold change > 2) and mapped to the human proteome using BLASTP against UniProtKB (*e*‐value < 1 × 10^−5^). Enrichment analyses for Gene Ontology (GO), Kyoto Encyclopedia of Genes and Genomes (KEGG) and WikiPathway pathways were conducted using clusterProfiler (v4.12.1). Detailed bioinformatic procedures and sequencing library preparation are provided in the Supporting Information.

### 2.8 Statistical Analysis

Statistical analyses were performed using R (v4.4.1). Normality was assessed using the Kolmogorov–Smirnov test. Continuous variables are presented as the mean ± standard deviation or median (interquartile range), and categorical variables as proportions. Group comparisons were conducted using nonparametric tests or the chi‐square test, as appropriate. Associations between central adiposity indices and musculoskeletal parameters were assessed using Spearman's correlation analyses. Multivariable binary logistic regression analyses were performed to examine independent associations, with results reported as odds ratios (ORs) and 95% confidence intervals (CIs). Receiver operating characteristic (ROC) curve analyses were used to determine the optimal cutoff values. In the longitudinal cohort, associations between anthropometric parameters and incident musculoskeletal disorders were evaluated using Cox proportional hazards models with time‐dependent covariates. The proportional hazards assumption was assessed using Schoenfeld residuals and was not violated. Results are reported as hazard ratios (HRs) with 95% CIs. All tests were two‐tailed; *p* < 0.05 was considered statistically significant.

## 3 Results

### 3.1 Cross‐Sectional Study

#### 3.1.1 Clinical Characteristics

The cross‐sectional study included 4157 patients with T2DM (2257 men and 1900 women) after age matching. Participants were categorised into four groups: non‐osteopenia and non‐sarcopenia (830 men, 619 women), osteopenia (400 men, 711 women), sarcopenia (501 men, 135 women) and SOs (526 men, 435 women), with a mean age of 59.4 ± 10.3 years.

Compared with the non‐osteopenia and non‐sarcopenia group, male patients with osteopenia, sarcopenia and SOs had significantly lower BMI. Patients with sarcopenia (with or without osteopenia) exhibited lower WC, WHtR and WHR than those without sarcopenia. WBR differed significantly across all male groups, showing a progressive increase from non‐osteopenia/non‐sarcopenia to osteopenia, sarcopenia and SOs. A similar pattern was observed in female participants. Detailed between‐group differences in biochemical parameters, body composition, comorbidities and medication use for both sexes are presented in Table 1.

**TABLE 1:** Baseline characteristics of the study participants.

	Male (2257)				Female (1900)			
	Non‐sarcopenia (1230)		Sarcopenia (1027)		Non‐sarcopenia (1330)		Sarcopenia (570)	
	Non‐osteopenia (830)	Osteopenia (400)	Non‐osteopenia (501)	Osteopenia (526)	Non‐osteopenia (619)	Osteopenia (711)	Non‐osteopenia (135)	Osteopenia (435)
Age (year)	59.08 ± 10.78	59.09 ± 11.30	59.63 ± 12.33	59.87 ± 10.72	58.83 ± 10.27	59.92 ± 7.46^a^	59.67 ± 11.40	59.76 ± 8.82^a^
Duration (year)	7.00 [2.00, 11.00]	7.00 [2.00, 11.25]	8.00 [2.00, 12.00]	7.00 [2.00, 12.00]	8.00 [2.00, 12.00]	8.00 [3.00, 12.00]	7.00 [1.50, 10.00]	7.00 [3.00, 10.00]
BMI (kg/m^2^)	26.65 ± 2.98	25.90 ± 2.84^a^	22.66 ± 2.33^a,b^	21.66 ± 2.52^a,b,c^	26.59 ± 3.95	24.91 ± 3.28^a^	21.58 ± 2.94^a,b^	21.10 ± 2.87^a,b^
WC (cm)	95.81 ± 12.32	94.01 ± 11.77	84.83 ± 9.71^a,b^	84.06 ± 10.75^a,b^	93.56 ± 16.28	89.16 ± 13.61^a^	78.55 ± 9.36^a,b^	81.39 ± 12.36^a,b^
WHtR (cm/cm)	0.57 ± 0.08	0.57 ± 0.08	0.51 ± 0.06^a,b^	0.51 ± 0.07^a,b^	0.60 ± 0.11	0.58 ± 0.09^a^	0.50 ± 0.06^a,b^	0.53 ± 0.08^a,b,c^
WHR (cm/cm)	0.98 ± 0.09	0.98 ± 0.09	0.93 ± 0.09^a,b^	0.93 ± 0.09^a,b^	0.96 ± 0.12	0.94 ± 0.10	0.88 ± 0.08^a,b^	0.93 ± 0.11^a,b,c^
WBR (cm * m^2^/kg)	3.59 ± 0.16	3.63 ± 0.13^a^	3.74 ± 0.12^a,b^	3.88 ± 0.13^a,b,c^	3.51 ± 0.19	3.57 ± 0.19^a^	3.65 ± 0.23^a,b^	3.85 ± 0.16^a,b,c^
HGB (g/L)	138.83 ± 18.72	136.27 ± 20.40	133.47 ± 20.73^a,b^	131.64 ± 21.29^a,b^	126.23 ± 16.53	125.33 ± 16.87	125.02 ± 15.48	122.14 ± 17.60^a,b^
ALB (g/L)	40.42 ± 4.82	39.61 ± 5.56	39.15 ± 4.83^a^	38.64 ± 4.78^a,b^	40.01 ± 4.51	39.94 ± 4.52	39.84 ± 4.87	39.15 ± 4.39^a,b^
ALT (U/L)	24.00 [17.25, 34.00]	24.00 [17.00, 32.25]	21.00 [14.00, 31.00]^a,b^	22.00 [16.00, 31.00]^a^	22.00 [15.00, 31.00]	19.00 [14.00, 26.50]^a^	20.00 [12.50, 25.50]	19.00 [13.00, 27.00]^a^
AST (U/L)	24.00 [18.00, 27.00]	23.00 [17.00, 27.00]	22.00 [16.00, 26.00]^a^	23.00 [17.00, 25.00]^a^	23.00 [17.00, 26.00]	21.00 [16.00, 25.00]^a^	21.00 [16.00, 24.00]^a^	21.00 [17.00, 24.50]^a^
eGFR (mL/[min/1.73m^2^])	93.43 ± 21.54	91.40 ± 26.33	94.74 ± 25.29	97.30 ± 25.32^a,b^	101.27 ± 17.32	97.93 ± 19.08^a^	104.63 ± 19.22^b^	101.43 ± 17.02^c^
UACR (mg/g)	14.48 [6.56, 92.48]	12.69 [6.34, 94.72]	14.19 [6.76, 98.05]	13.68 [6.45, 71.83]	16.99 [8.11, 108.21]	17.05 [7.52, 93.08]	13.92 [6.31, 78.56]	19.62 [8.45, 120.20]
TCH (mmol/L)	4.48 ± 1.15	4.56 ± 1.28	4.44 ± 1.23	4.42 ± 1.39	4.88 ± 1.25	4.89 ± 1.24	4.90 ± 1.55	4.81 ± 1.21
TG (mmol/L)	1.61 [1.10, 2.28]	1.45 [0.97, 2.16]^a^	1.31 [0.94, 1.86]^a^	1.22 [0.85, 1.78]^a,b^	1.71 [1.16, 2.33]	1.59 [1.10, 2.26]	1.36 [1.00, 1.79]^a,b^	1.41 [0.94, 2.00]^a,b^
HDL‐c (mmol/L)	1.03 ± 0.29	1.06 ± 0.37	1.07 ± 0.32^a^	1.09 ± 0.32^a,b^	1.16 ± 0.32	1.21 ± 0.35	1.22 ± 0.31^a^	1.27 ± 0.37^a,b^
LDL‐c (mmol/L)	2.81 ± 1.05	2.93 ± 1.07	2.87 ± 1.17	2.93 ± 1.37	3.05 ± 1.14	3.04 ± 1.13	3.09 ± 1.23	2.97 ± 1.07
HBA1c (%)	9.14 ± 2.30	9.24 ± 2.32	9.58 ± 2.54^a^	9.63 ± 2.77^a^	9.07 ± 2.10	8.97 ± 2.36	9.66 ± 2.50^b^	9.21 ± 2.48
25(OH)D (ng/ml)	25.62 ± 9.23	23.53 ± 9.89^a^	24.99 ± 9.60	23.34 ± 9.33^a,c^	23.59 ± 8.43	22.81 ± 8.16	23.19 ± 7.91	23.57 ± 8.42
Ca (mmol/L)	2.23 ± 0.12	2.21 ± 0.15	2.23 ± 0.14	2.21 ± 0.13^a^	2.25 ± 0.13	2.24 ± 0.13	2.26 ± 0.14	2.25 ± 0.13
L_1 ~ 4_ BMD (g/cm^2^)	1.26 ± 0.16	1.05 ± 0.15^a^	1.23 ± 0.15^a,b^	1.01 ± 0.15^a,b,c^	1.19 ± 0.16	0.93 ± 0.12^a^	1.18 ± 0.18^b^	0.88 ± 0.14^a,b,c^
LFN BMD (g/cm^2^)	1.01 ± 0.11	0.82 ± 0.11^a^	0.99 ± 0.11^a,b^	0.79 ± 0.12^a,b,c^	1.00 ± 0.13	0.80 ± 0.13^a^	0.97 ± 0.11^b^	0.74 ± 0.13^a,b,c^
LH BMD (g/cm^2^)	1.09 ± 0.11	0.90 ± 0.11^a^	1.06 ± 0.11^a,b^	0.86 ± 0.13^a,b,c^	1.07 ± 0.12	0.86 ± 0.13^a^	1.03 ± 0.10^a,b^	0.80 ± 0.14^a,b,c^
L_1 ~ 4_, T	1.10 ± 1.40	−0.64 ± 1.28^a^	0.91 ± 1.29^b^	−1.01 ± 1.39^a,b,c^	0.38 ± 1.06	−1.80 ± 1.06^a^	0.36 ± 1.31^b^	−2.24 ± 1.24^a,b,c^
LFN, T	−0.04 ± 0.72	−1.53 ± 0.71^a^	−0.15 ± 0.69^a,b^	−1.72 ± 0.81^a,b,c^	0.25 ± 0.83	−1.44 ± 0.95^a^	0.04 ± 0.80^a,b^	−1.97 ± 1.07^a,b,c^
LH, T	0.67 ± 0.80	−0.72 ± 0.84^a^	0.48 ± 0.79^a,b^	−1.07 ± 1.00^a,b,c^	0.66 ± 0.84	−0.91 ± 0.99^a^	0.34 ± 0.75^a,b^	−1.45 ± 1.10^a,b,c^
ASMI (kg/m^2^)	8.17 ± 0.89	8.01 ± 0.67^a^	6.63 ± 0.50^a,b^	6.46 ± 0.62^a,b,c^	6.73 ± 0.83	6.46 ± 0.78^a^	5.28 ± 0.30^a,b^	5.18 ± 0.42^a,b^
BF (%)	25.95 ± 5.97	24.41 ± 7.08^a^	24.32 ± 6.57^a^	22.36 ± 7.38^a,b,c^	34.35 ± 6.12	32.84 ± 6.74^a^	31.53 ± 6.16^a^	30.95 ± 7.39^a,b^
SOb, *N* (%)	—	—	225 (44.9%)	201 (38.2%)	—	—	39 (28.9%)	131 (30.1%)
Fracture, *N* (%)	28 (3.4%)	25 (6.2%)	19 (3.8%)	38 (7.2%)^a^	27 (4.4%)	51 (7.2%)	6 (4.4%)	31 (7.1%)
MOF (%)	1.70 [1.40, 2.10]	3.00 [2.48, 3.70]^a^	1.80 [1.40, 2.20]^b^	3.10 [2.50, 3.90]^a,c^	2.40 [1.80, 3.00]	4.00 [3.10, 5.05]^a^	2.50 [1.80, 3.10]^b^	4.50 [3.60, 5.50]^a,b,c^
HF (%)	0.30 [0.10, 0.53]	1.10 [0.70, 1.56]^a^	0.34 [0.10, 0.60]^a,b^	1.21 [0.84, 1.77]^a,b,c^	0.17 [0.00, 0.40]	1.07 [0.60, 1.64]^a^	0.21 [0.04, 0.60]^a,b^	1.50 [0.90, 2.16]^a,b,c^
DR, *N* (%)	177 (21.3%)	96 (24.0%)	105 (21.0%)	128 (24.3%)	152 (24.6%)	174 (24.5%)	30 (22.2%)	92 (21.1%)
DKD, *N* (%)	277 (33.4%)	135 (33.8%)	173 (34.5%)	169 (32.1%)	205 (33.1%)	223 (31.4%)	42 (31.1%)	149 (34.3%)
DPN, *N* (%)	453 (54.6%)	219 (54.8%)	286 (57.1%)	299 (56.8%)	354 (57.2%)	397 (55.8%)	75 (55.6%)	216 (49.7%)
Hypertension, *N* (%)	415 (50.0%)	212 (53.0%)	207 (41.3%)^a,b^	213 (40.5%)^a,b^	303 (48.9%)	340 (47.8%)	41 (30.4%)^a,b^	155 (35.6%)^a,b^
Smoking, *N* (%)	332 (40.0%)	178 (44.5%)	187 (37.3%)	253 (48.1%)^a,c^	6 (1.0%)	3 (0.4%)	0 (0.0%)	0 (0.0%)
Drinking, *N* (%)	147 (17.7%)	68 (17.0%)	75 (15.0%)	91 (17.3%)	4 (0.6%)	2 (0.3%)	0 (0.0%)	1 (0.2%)
Exercise (min/week)	29.87 ± 5.48	29.86 ± 5.51	29.61 ± 5.84	29.84 ± 5.79	30.53 ± 5.37	29.32 ± 5.07	30.52 ± 5.64	29.95 ± 5.27
Biguanide, *N* (%)	531 (64.0%)	243 (60.8%)	289 (57.7%)	275 (52.3%)^a^	428 (69.1%)	436 (61.3%)^a^	78 (57.8%)	241 (55.4%)^a^
SGLT‐2i, *N* (%)	247 (29.8%)	119 (29.8%)	141 (28.1%)	149 (28.3%)	155 (25.0%)	168 (23.6%)	30 (22.2%)	95 (21.8%)
Other GLDs, *n* (%)	572 (68.9%)	275 (68.8%)	352 (70.3%)	350 (66.5%)	451 (72.9%)	523 (73.6%)	99 (73.3%)	309 (71.0%)
Insulin, *N* (%)	235 (28.3%)	129 (32.2%)	139 (27.7%)	166 (31.6%)	203 (32.8%)	210 (29.5%)	46 (34.1%)	130 (29.9%)
Antihypertensive medications, *N* (%)	340 (41.0%)	180 (45.0%)	174 (34.7%)^b^	175 (33.3%)^a,b^	230 (37.2%)	289 (40.6%)	30 (22.2%)^a,b^	113 (26.0%)^a,b^
Lipid‐lowering medications, *N* (%)	223 (26.9%)	83 (20.8%)	107 (21.4%)	95 (18.1%)^a^	116 (18.7%)	147 (20.7%)	25 (18.5%)	67 (15.4%)
Anti‐osteoporotic medications, *N* (%)	—	2 (0.5%)	—	3 (0.6%)	—	5 (0.7%)	—	8 (1.8%)

*Note:* Compared with the non‐osteopenia/non‐sarcopenia group, *p* < 0.05 is denoted by superscript a; compared with the osteopenia group, *p* < 0.05 is denoted by superscript b; compared with the sarcopenia group, *p* < 0.05 is denoted by superscript c. Values are presented as mean ± standard deviation, median (interquartile range) or number (percentage), as appropriate. Anti‐osteoporotic medication use was minimal at baseline, as most participants were newly diagnosed and had not initiated osteoporosis‐specific treatment.

Abbreviations: 25(OH)D, 25‐hydroxyvitamin D; ALB, albumin; ALT, alanine aminotransferase; ASMI, appendicular skeletal muscle mass index; AST, aspartate aminotransferase; BMD, bone mineral density; BF, body fat percentage; BMI, body mass index; DKD, diabetic kidney disease; DPN, diabetic peripheral neuropathy; DR, diabetic retinopathy; GLDs, glucose‐lowering drugs; HbA1c, glycated haemoglobin; HDL‐C, high‐density lipoprotein cholesterol; HF, hip fracture probability; HGB, haemoglobin; L1–4, lumbar spine (L1–L4); LDL‐C, low‐density lipoprotein cholesterol; LFN, left femoral neck; LH, left hip; MOF, major osteoporotic fracture probability; SGLT‐2i, sodium–glucose cotransporter‐2 inhibitors; SOb, sarcopenic obesity; T, T‐score; TCH, total cholesterol; TG, triglycerides; UACR, urinary albumin‐to‐creatinine ratio; WBR, weight‐to‐body surface area ratio; WC, waist circumference; WHR, waist‐to‐hip ratio; WHtR, waist‐to‐height ratio.

#### 3.1.2 Correlations Between Anthropometric Indices and Musculoskeletal Parameters

**FIGURE 3:**
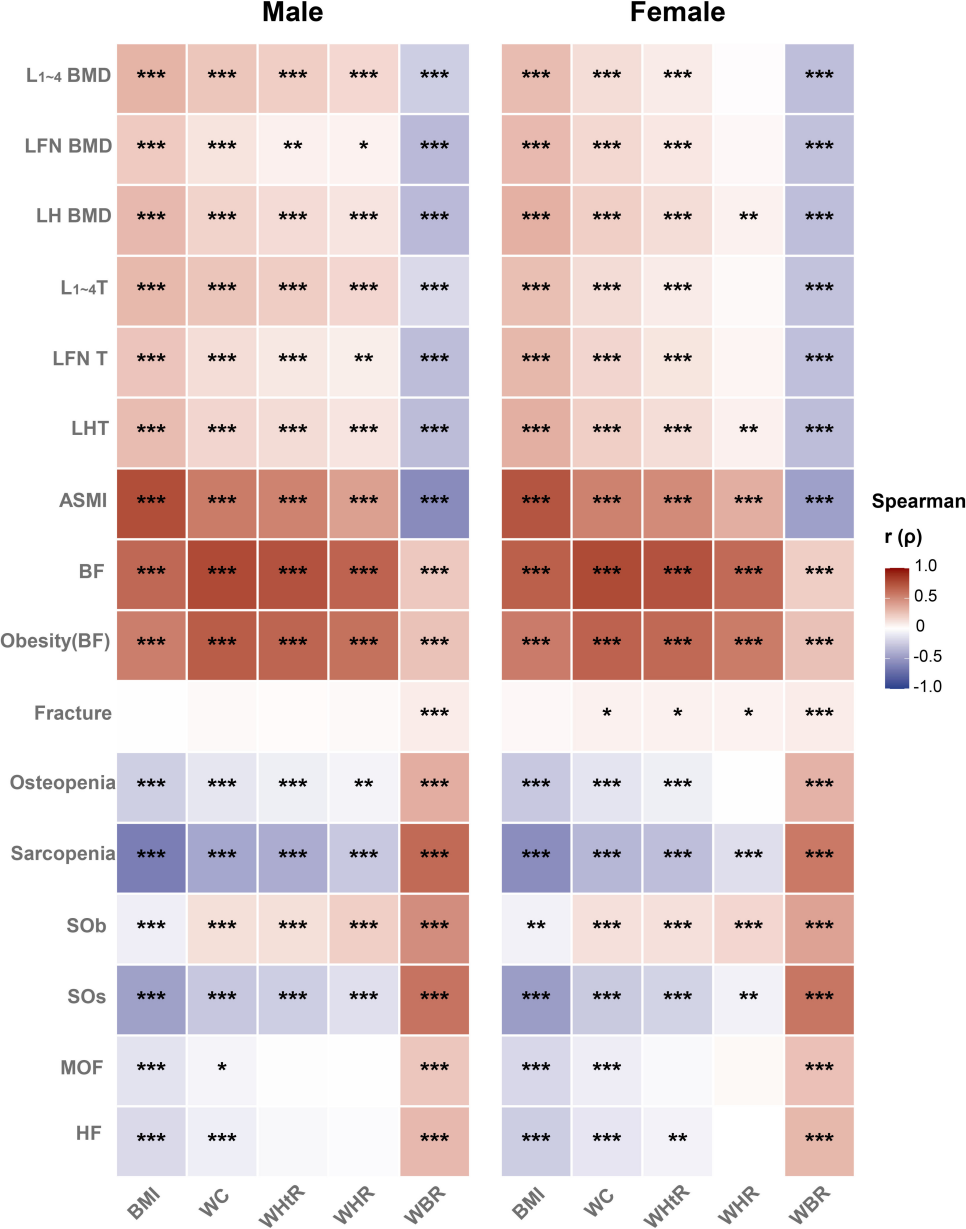
Heatmap of correlations between waist‐related anthropometric indices and musculoskeletal parameters Correlations between waist circumference–related indices and BMD, T‐score, ASMI, BF, fracture risk and musculoskeletal outcomes are shown separately for males and females. *0.01 ≤ *p* < 0.05; **0.001 ≤ *p* < 0.01; ***p < 0.001.

WBR showed significant negative correlations with BMD, T‐score and ASMI and significant positive correlations with BF, fracture, osteopenia, sarcopenia, obesity, SOb, SOs, major osteoporotic fracture probability (MOF) and hip fracture probability (HF) (all *p* < 0.01). BMI showed positive correlations with BMD, T‐score, ASMI and BF and negative correlations with sarcopenia‐related indicators, while its associations with fracture events were weak. WC, WHtR and WHR exhibited weak or nonsignificant correlations with musculoskeletal indicators and clinical events (Figure 3).

**FIGURE 4:**
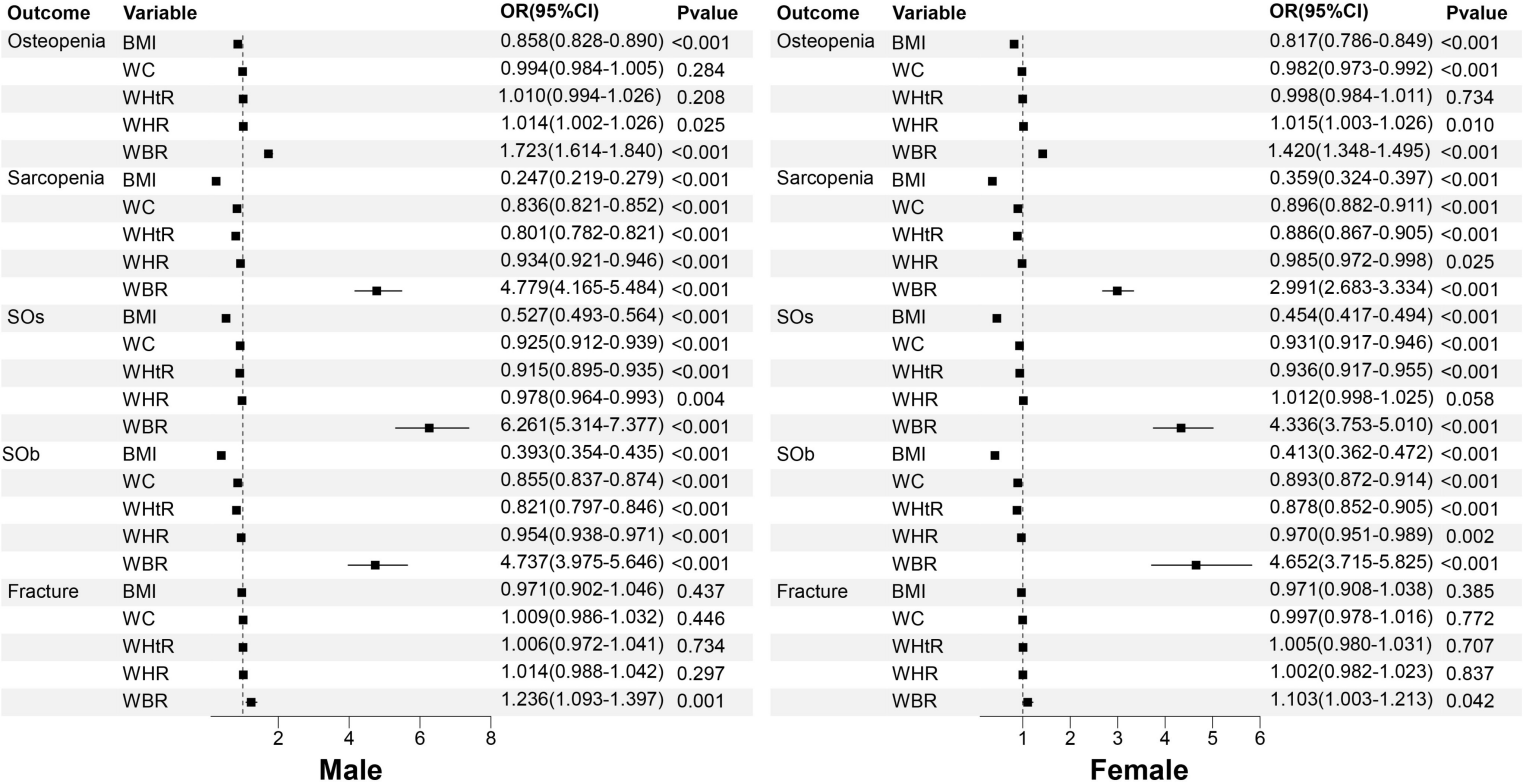
Associations of BMI, WC, WHtR, WHR and WBR with musculoskeletal diseases based on binary logistic regression analyses. Binary logistic regression analyses were performed to evaluate the associations of BMI, WC, WHtR, WHR and WBR with various musculoskeletal diseases. Models were adjusted for age, BF, duration of diabetes mellitus, diabetic complications (DR, DKD and DPN), smoking status, drinking status, exercise, haemoglobin, albumin, UACR, LDL‐C, HbA1c, Ca, vitamin D status and antidiabetic medications (biguanides, SGLT2i and insulin therapy). Because WHtR, WHR and WBR are ratio‐based indices with small unit increments, rescaling (WHtR × 100, WHR × 100 and WBR × 10) was applied prior to regression analyses to facilitate interpretation of ORs without altering the underlying model or statistical significance. Vitamin D status (25‐hydroxyvitamin D, ng/mL): insufficient (≤30 ng/mL), sufficient (> 30 ng/mL).

WBR was independently associated with musculoskeletal outcomes in patients with T2DM. WBR was independently associated with osteopenia (men: OR 1.723, 1.614–1.840; women: OR 1.420, 1.348–1.495), sarcopenia (men: OR 4.779, 4.165–5.484; women: OR 2.991, 2.683–3.334) and SOs (men: OR 6.261, 5.314–7.377; women: OR 4.336, 3.753–5.010). WBR was also independently associated with SOb (men: OR 4.737, 3.975–5.646; women: OR 4.652, 3.715–5.825) and fracture risk (men: OR 1.236, 1.093–1.397; women: OR 1.103, 1.003–1.213). All associations between WBR and musculoskeletal outcomes were statistically significant (all *p* < 0.05). BMI and other anthropometric indices generally showed weaker or nonsignificant associations with musculoskeletal outcomes, particularly with respect to fracture risk (Figure 4). Detailed subgroup analyses are presented in Figures [Link], [Link], [Link].

**FIGURE 5:**
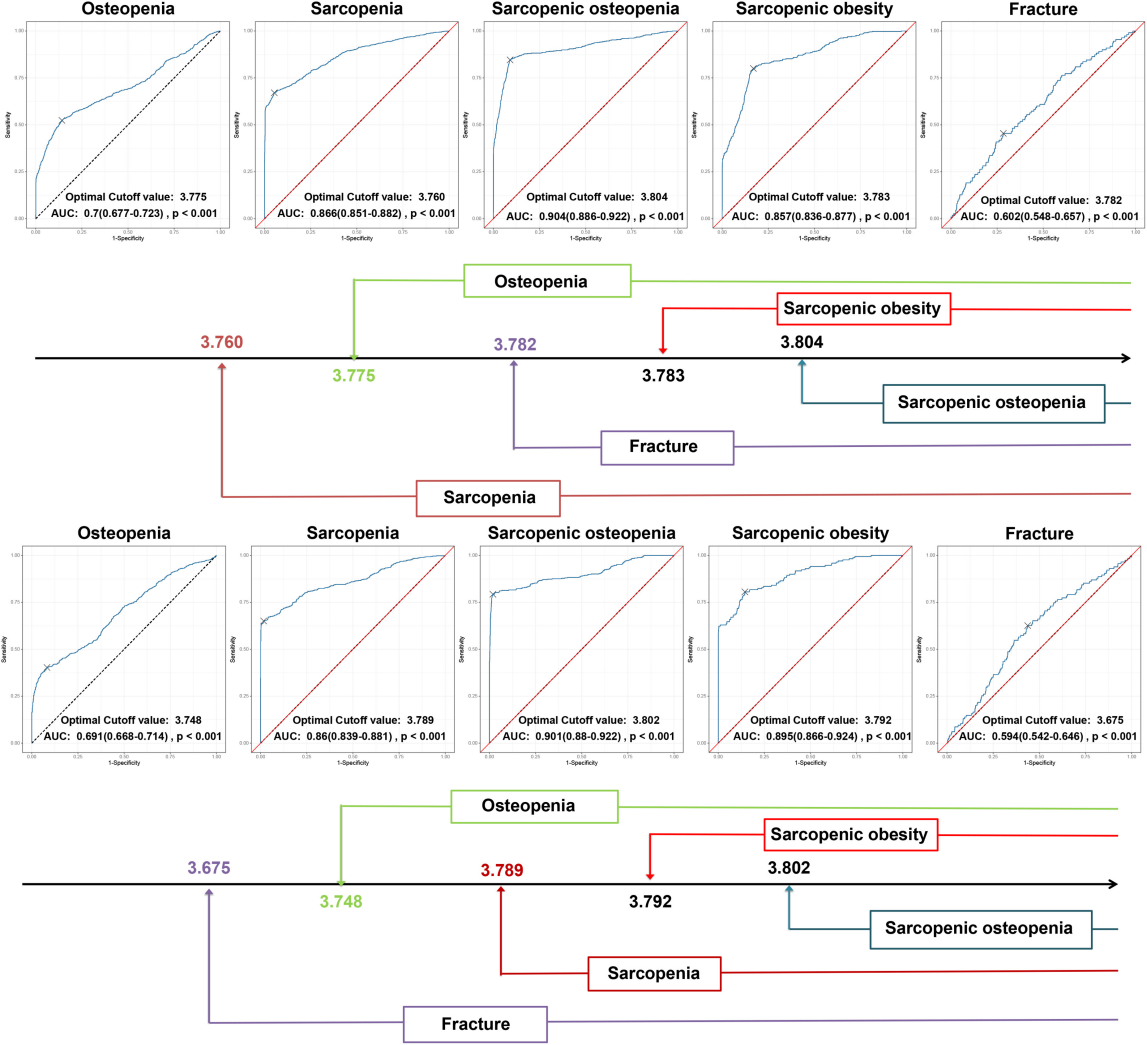
ROC analysis of WBR for musculoskeletal outcomes in patients with T2DM. ROC curves of WBR for osteopenia, sarcopenia, sarcopenic osteopenia, fracture and sarcopenic obesity in patients with type 2 diabetes mellitus. Results are presented separately for male (upper panels) and female (lower panels) participants. The AUC, 95% confidence intervals (95% CI) and *p* values are shown within each ROC plot. The diagonal reference line represents no discrimination. The lower panels illustrate the corresponding WBR cutoff values for each outcome, as derived from the ROC analysis, displayed along the WBR axis for males and females.

The optimal WBR cutoffs for discriminating musculoskeletal outcomes were determined by ROC curve analysis (all *p* < 0.001; Figure 5 and Figure S4). In men, the cutoffs (AUC, 95% CI) were as follows: osteopenia 3.775 (0.670, 0.617–0.723), fracture 3.782 (0.602, 0.548–0.657), sarcopenia 3.760 (0.866, 0.851–0.882), SOs 3.804 (0.904, 0.886–0.922) and SOb 3.783 (0.857, 0.836–0.877). In women, the corresponding values were as follows: osteopenia 3.748 (0.691, 0.668–0.714), sarcopenia 3.789 (0.860, 0.839–0.881), fracture 3.675 (0.594, 0.542–0.646), SOs 3.802 (0.901, 0.880–0.922) and SOb 3.792 (0.895, 0.868–0.924).

### 3.2 Follow‐Up Study

**FIGURE 6:**
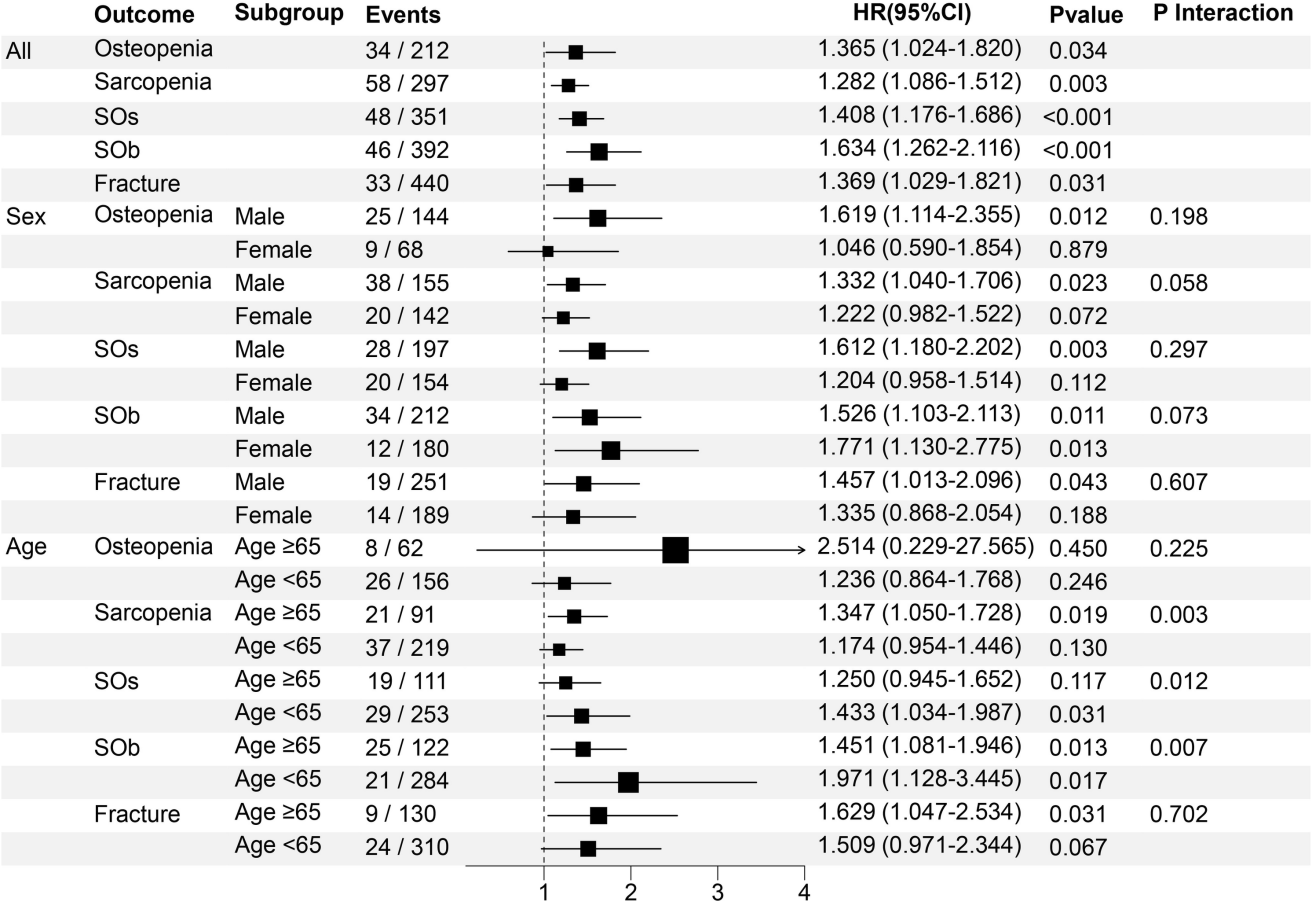
Time‐dependent Cox regression analysis of WBR in relation to musculoskeletal outcomes. Forest plot of HRs and 95% CIs for the associations between WBR and osteopenia, sarcopenia, sarcopenic osteopenia, sarcopenic obesity and fracture, estimated using time‐dependent Cox regression models. To facilitate interpretation of effect estimates, WBR was rescaled (WBR × 10) prior to modelling; this rescaling does not affect the underlying model fit or statistical significance. Multivariable models were adjusted for sex, age, diabetes duration, smoking status, drinking status, vitamin D status, DR, DKD, DPN, HbA1c, use of biguanides, insulin therapy and SGLT‐2i. In sex‐stratified analyses, sex was not included as an adjustment variable; in age‐stratified analyses, age was not included as an adjustment variable. Squares represent point estimates of HRs, horizontal lines indicate 95% CIs and the vertical dashed line denotes the reference value (HR = 1). *p* values and *p* values for interaction are shown for subgroup analyses. For each outcome, participants with the corresponding musculoskeletal condition present at baseline were excluded from the analysis; for fracture outcomes, participants were not excluded at baseline because fractures can recur.

This prospective cohort study included 440 patients with T2DM (251 men, 189 women) with complete follow‐up data. The mean age was 59.72 ± 9.74 years, and the median follow‐up duration was 34.00 months (interquartile range [IQR], 20.00–57.00 months). At baseline, the median duration of diabetes was 10.00 years (IQR, 4.00–13.00 years). Metabolic and biochemical characteristics are summarised in Table S1. Higher WBR was independently associated with an increased risk of adverse musculoskeletal outcomes during follow‐up (Figure 6). In the overall cohort, higher time‐dependent WBR was associated with a significantly higher risk of incident osteopenia (HR 1.365, 1.024–1.820; *p* = 0.034), sarcopenia (HR 1.282, 1.086–1.512; *p* = 0.003), SOs (HR 1.408, 1.176–1.686; *p* < 0.001), SOb (HR 1.634, 1.262–2.116; p < 0.001) and incident fracture (HR 1.369, 1.029–1.821; *p* = 0.031).

Sex‐stratified analyses showed that higher WBR was associated with increased risks of osteopenia (HR 1.619, 1.114–2.355; *p* = 0.012), sarcopenia (HR 1.332, 1.040–1.706; *p* = 0.023), SOs (HR 1.612, 1.180–2.202; *p* = 0.003) and fracture (HR 1.457, 1.013–2.096; *p* = 0.043) in men, whereas most associations in women were not statistically significant. For SOb, significant associations were observed in both men (HR 1.526, 1.103–2.113; *p* = 0.011) and women (HR 1.771, 1.130–2.775; *p* = 0.013). No significant sex interactions were detected (all p for interaction > 0.05).

Age‐stratified analyses showed that higher WBR was associated with sarcopenia in participants aged ≥ 65 years (HR 1.347, 1.050–1.728; *p* = 0.019) and with SOs in those aged < 65 years (HR 1.433, 1.034–1.987; *p* = 0.031), with significant age interactions for both outcomes. For SOb, significant associations were observed in both age groups (≥65 years: HR 1.451, 1.081–1.946; *p* = 0.013; <65 years: HR 1.971, 1.128–3.445; *p* = 0.017), with evidence of interaction. Higher WBR was also associated with fracture among participants aged ≥65 years (HR 1.629, 1.047–2.534; *p* = 0.031), whereas no significant age interaction was detected. No interaction was observed for osteopenia.

### 3.3 Autoantibody Profiling Analysis

Because the primary analyses modelled WBR as a time‐dependent exposure, grouping participants by baseline WBR would not adequately capture long‐term WBR trajectories. Given the cross‐sectional nature of autoantibody profiling, participants were stratified by extreme values of ΔWBR/yr, representing non‐increase and increase patterns over follow‐up.

**FIGURE 7:**
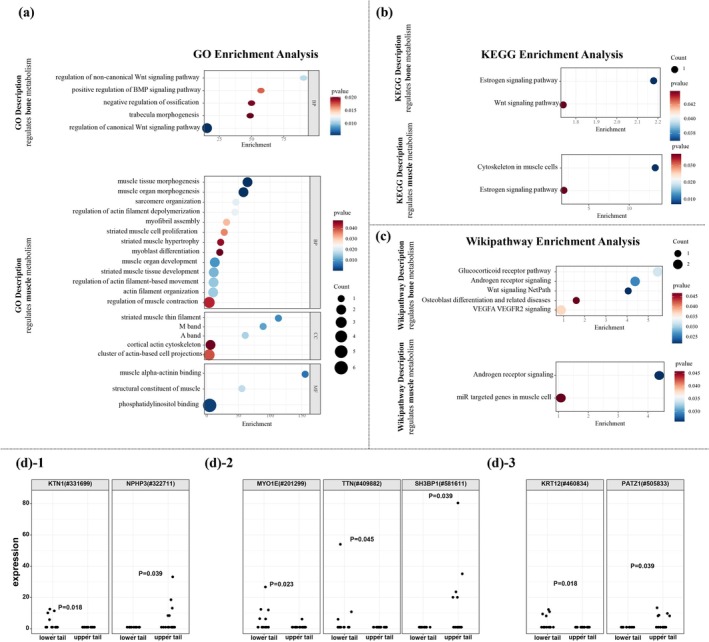
Autoantibody profiling and pathway enrichment analysis according to annualised WBR change (ΔWBR/yr). Autoantibody profiling analyses comparing participants stratified by extreme values of annualised waist‐to‐body ratio change (ΔWBR/yr), representing non‐increase and increase patterns during follow‐up. (a) GO enrichment analysis of differentially reactive autoantibody targets, including biological processes, cellular components and molecular functions related to bone and muscle metabolism. (b) KEGG pathway enrichment analysis highlighting pathways associated with bone and muscle metabolism. (c) Wikipathway enrichment analysis identifying regulatory pathways involved in skeletal and muscle metabolic processes. Dot size indicates the number of enriched targets, and colour represents the enrichment *p* value. (d) Differential autoantibody reactivity for selected proteins associated with (d‐1) bone metabolism, (d‐2) muscle metabolism and (d‐3) both bone and muscle metabolism between the ΔWBR/yr non‐increase and increase groups. Individual points represent participants, and *p* values indicate between‐group differences.

GO enrichment revealed significant differences in autoantibody repertoires between the ΔWBR/yr non‐increase and increase groups across biological processes, cellular components and molecular functions related to muscle development, whereas only biological processes showed significant enrichment for bone development. KEGG pathway analysis indicated that intergroup differences in muscle formation were associated with cytoskeletal organisation, while bone metabolism disparities were primarily mediated by the Wnt signalling pathway. The oestrogen signalling pathway regulated both bone and muscle metabolism. Wikipathway analysis further suggested potential miRNA‐mediated regulation of muscle metabolism, while bone metabolism involved the Wnt signalling network, glucocorticoid receptor pathway, vascular endothelial growth factor (VEGF)A‐VEGF receptor 2 signalling and osteoblast differentiation processes. The androgen pathway demonstrated regulatory roles in both skeletal and muscle metabolism. Significant differences in autoantibody reactivity (*p* < 0.05) were observed for skeletal metabolism targets (KTN1, NPHP3), muscle metabolism targets (MYO1E, TTN, SH3BP1) and musculoskeletal metabolism targets (KRT12, PATZ1) between the two groups (Figure 7).

## 4 Discussion

WBR was identified as a key anthropometric marker associated with skeletal muscle complications in patients with T2DM. WBR values were progressively higher across phenotypic groups and demonstrated better discriminative performance than traditional indices (WC, WHtR and WHR). Higher WBR levels were associated with an increased risk of osteopenia or sarcopenia and earlier onset of these conditions. These findings support WBR as a novel, non‐invasive and primary‐care‐accessible tool for monitoring skeletal muscle health in patients with T2DM.

Recent studies have questioned the adequacy of conventional anthropometric indices in capturing adverse body‐composition phenotypes relevant to musculoskeletal health. Cho et al. reported that WHtR–derived fat mass categories were associated with incident fractures, highlighting the contribution of central adiposity beyond BMI [14]. However, that study was conducted in a general population and did not assess muscle‐related phenotypes or individuals with T2DM, a group characterised by altered fat distribution, insulin resistance and increased fracture risk despite preserved or even elevated BMD. Meta‐analyses have further demonstrated heterogeneous and site‐specific associations between BMI and fracture risk, challenging the long‐held assumption that higher BMI is uniformly protective [22]. In addition, observational studies have shown that BMI fails to distinguish fat from lean mass and does not capture ectopic or visceral adiposity [2, 3], limiting its relevance in metabolically compromised populations [4, 10]. Although WC, WHtR and WHR have been proposed as surrogate markers of central obesity, their associations with sarcopenia and combined osteo‐sarcopenic phenotypes have been inconsistent, and most prior studies have evaluated single indices in isolation rather than performing head‐to‐head comparisons. Our study simultaneously evaluated multiple anthropometric parameters within a well‐characterised T2DM cohort and demonstrated that WBR showed stronger and more consistent associations with osteopenia, sarcopenia, SOb and fractures than conventional indices. Importantly, we extended prior cross‐sectional findings by incorporating longitudinal analyses with time‐dependent modelling, demonstrating that increases in WBR over time were associated with incident musculoskeletal outcomes. These findings suggest that integrating central adiposity with overall body mass captures adverse body‐composition features relevant to T2DM and may provide incremental prognostic information beyond traditional anthropometric measures.

Follow‐up of selected patients with diabetes revealed that elevated WBR was associated with increased risks of sarcopenia, osteopenia, SOb, SOs and fractures in T2DM. These associations likely arise from multifactorial mechanisms. Elevated WBR likely reflects visceral adipose tissue expansion, which disrupts muscle and bone metabolism through chronic inflammation and metabolic dysregulation [23, 24]. VAT‐derived cytokines and adipokines promote muscle protein degradation, impair myogenesis and enhance osteoclast activity while suppressing osteoblast function [25, 26]. Increased free fatty acid flux contributes to systemic and skeletal‐muscle insulin resistance [27], linking adipose tissue dysfunction to impaired PI3K/Akt–mTOR signalling and reduced muscle protein synthesis [23]. In T2DM, chronic hyperglycaemia and AGE accumulation further exacerbate muscle stiffness and bone fragility through oxidative stress and osteocyte apoptosis [8, 28, 29]. Collectively, these mechanisms provide a plausible biological basis for the association between higher WBR and musculoskeletal deterioration.

This study identified sex‐specific WBR cutoff values across different musculoskeletal outcomes, with relatively narrow ranges and partial overlap between conditions. Although the cutoffs did not follow a strictly monotonic sequence, modest variations in thresholds were observed across phenotypes. These patterns may reflect sex‐specific differences in body composition and vulnerability to musculoskeletal complications. Given the clustering of thresholds within a limited range, even small changes in WBR may correspond to meaningful differences in musculoskeletal risk. While the discriminative performance of these cutoffs supports the potential utility of WBR in risk stratification, the proximity of thresholds warrants cautious interpretation.

Pathway enrichment analysis of PhIP‐Seq‐derived antibody reactivity profiles revealed distinct regulatory patterns between lower and higher WBR groups. Muscle‐related differences were predominantly enriched in structural and cytoskeletal pathways, suggesting alterations in contractile architecture and functional integrity. In contrast, skeletal differences were primarily associated with signalling cascades rather than structural components. Enrichment of Wnt and BMP signalling pathways provides mechanistic plausibility, given their established roles in osteogenic differentiation and bone metabolism [30]. Additional involvement of glucocorticoid receptor signalling [31] and the VEGFA–VEGF receptor 2 pathway [32] highlights potential perturbations in bone–vascular coupling and remodelling dynamics. The concurrent enrichment of oestrogen and androgen signalling pathways [33, 34] suggests hormonal modulation as a shared regulatory axis across bone and muscle tissues, while miRNA‐related pathways [35] point to possible post‐transcriptional regulation. These findings should be interpreted as hypothesis‐generating rather than confirmatory, but they support the concept that WBR may reflect integrated disturbances across interconnected bone–muscle regulatory networks.

WBR is a noninvasive and reproducible anthropometric index derived from weight, height and WC. Compared with imaging‐based assessments such as DXA or CT, it is inexpensive and readily applicable in primary care. Musculoskeletal risk assessment in T2DM largely relies on BMI, DXA and FRAX, yet BMI does not adequately reflect adverse body composition. Incorporating WBR into routine diabetes care may therefore help identify high‐risk individuals who could otherwise be overlooked. Although external validation is required, WBR may serve as a practical adjunctive screening tool in clinical practice. The observed sex‐specific differences further suggest heterogeneity in risk patterns and warrant further investigation.

## 5 Limitations

First, the cross‐sectional component precludes causal inference, and retrospective fracture ascertainment may have preceded exposure assessment. Although longitudinal analyses were performed, the overall observational design does not eliminate residual confounding. Second, the cohort was derived from a single tertiary centre with predominantly Asian participants, which may limit generalisability. The longitudinal subset was modest in size; the median follow‐up of 34 months may be insufficient for long‐term fracture assessment, and the limited number of incident fractures warrants cautious interpretation of HRs. Third, WBR is an indirect anthropometric surrogate. Variability in waist measurement may introduce error, and WBR cannot distinguish visceral from subcutaneous fat or assess muscle quality. Sarcopenia was defined using DXA‐derived muscle mass without direct measures of strength or performance. Fourth, despite multivariable adjustment, unmeasured confounders, including diet, physical activity, bone‐active medications and sex hormones, may have influenced the results. Finally, the PhIP‐Seq analysis was exploratory and based on a small extreme‐phenotype subset; findings are hypothesis‐generating and require external validation.

## 6 Future Directions

Future studies should include large, multicentre prospective cohorts with longer follow‐up to validate the prognostic utility of WBR across diverse populations. External validation of the proposed sex‐specific cut‐off values is warranted. Integration of imaging‐based adiposity assessment, muscle strength evaluation and functional performance testing would help refine risk stratification. Mechanistic studies are needed to further elucidate the biological pathways linking WBR to musculoskeletal deterioration in T2DM.

## 7 Conclusion

Higher WBR levels and longitudinal increases were independently associated with musculoskeletal disorders and fractures in individuals with T2DM. Compared with traditional anthropometric indices, WBR showed stronger associations with adverse bone–muscle outcomes.

## Funding

This study was supported by grants from the National Natural Science Foundation of China (grant numbers 82300984 and 82371601) and the Scientific and Technological Major Special Project of Fujian Provincial Health Commission (No. 2021ZD01004).

## Ethics Statement

This study was approved by the Ethics Committee of the First Affiliated Hospital of Fujian Medical University (Approval Nos. 2011.04, 2012.11 and 2017.131). All procedures were conducted in accordance with the ethical standards of the institutional and national research committees and with the Declaration of Helsinki (1975, as revised in 2013). Written informed consent was obtained from all participants. All authors of this manuscript comply with the guidelines of ethical authorship and publishing in the *Journal of Cachexia, Sarcopenia and Muscle*.

## Consent

The authors have nothing to report.

## Conflicts of Interest

The authors declare no conflicts of interest.

## Supporting information


**DATA S1:** Supplementary Information.

**DATA S2:** Supplementary Information.

**DATA S3:** Supplementary Information.

**FIGURE S1:** Subgroup analyses stratified by duration of diabetes mellitus in men and women. Subgroup analyses were conducted to examine the associations of BMI, WC, WHtR, WHR and WBR with musculoskeletal diseases stratified by duration of diabetes mellitus, separately in men and women. Among men, the numbers of participants with a diabetes duration of ≤ 5 years, 5–15 years and > 15 years were 967, 967 and 323, respectively. Among women, the corresponding numbers were 757, 877 and 266, respectively. Binary logistic regression models were adjusted for age, BF, duration of diabetes mellitus, diabetic complications (DR, DKD and DPN), smoking status, drinking status, exercise, haemoglobin, albumin, UACR, LDL‐C, HbA1c, Ca, vitamin D status and antidiabetic medications (biguanides, SGLT2i and insulin therapy).

**FIGURE S2:** Subgroup analyses stratified by menopausal status in female participants. Subgroup analyses were performed to assess the associations of BMI, WC, WHtR, WHR and WBR with musculoskeletal diseases among female participants stratified by menopausal status. Among female participants, 312 were premenopausal and 1588 were postmenopausal. Binary logistic regression models were adjusted for age, BF, duration of diabetes mellitus, diabetic complications (DR, DKD and DPN), smoking status, drinking status, exercise, haemoglobin, albumin, UACR, LDL‐C, HbA1c, Ca, vitamin D status and antidiabetic medications (biguanides, SGLT2i and insulin therapy).

**FIGURE S3:** Sensitivity analyses excluding participants with hysterectomy. Sensitivity analyses were conducted after excluding participants with a history of hysterectomy (*n* = 55) because hysterectomy represents a heterogeneous clinical condition with respect to surgical indications and postoperative hormonal status, which could not be adequately characterised in the present study. The resulting analytical sample included 1845 women without hysterectomy. The associations of BMI, WC, WHtR, WHR and WBR with musculoskeletal diseases were re‐evaluated using binary logistic regression models. Models were adjusted for age, BF, DR, DKD, DPN, smoking status, drinking status, exercise, haemoglobin, albumin, UACR, LDL‐C, HbA1c, Ca, vitamin D status and antidiabetic medications (biguanides, SGLT2i and insulin therapy).

**FIGURE S4:** ROC curves of anthropometric indices for musculoskeletal conditions in men and women. ROC curves were used to compare the discriminative performance of BMI, WC, WHtR, WHR and WBR for identifying osteopenia, sarcopenia, sarcopenic osteopenia, fracture and sarcopenic obesity. Analyses were performed separately in men and women. AUC, 95% confidence interval (CI) and corresponding *p* values are shown for each anthropometric index. Higher AUC values indicate better discriminative ability.

## Data Availability

The datasets generated and/or analysed during this study are not publicly available due to concerns regarding participant/patient anonymity. Requests to access the datasets should be directed to the corresponding author. Detailed omics data and full specifications of the logistic regression and time‐dependent Cox (TD‐Cox) regression models are provided in the Supporting Information.
