# Supplementary material for: Waist‐to‐Body Mass Index Ratio and Risk of Musculoskeletal Disease in Type 2 Diabetes: A Cohort and PhIP‐Seq Analysis
Source: J Cachexia Sarcopenia Muscle. 2026 Aug 7;17(4):e70336. doi: 10.1002/jcsm.70336 (PMC13448775; doi:10.1002/jcsm.70336)
Supplement: Supplementary file 1 [file JCSM-17-e70336-s004.docx]

**Supplementary Methods: PhIP‑Seq Experimental Procedures and Bioinformatic Processing**

**2.7. Autoantibody Profiling by Phage Immunoprecipitation Sequencing**

**2.7.1. Phage Immunoprecipitation Assay**

For each serum sample, 4 μL of 100-fold diluted serum was combined with 100 μL of immunoprecipitation mixture. Control reactions were established by adding 100 μL of immunoprecipitation mixture to 1 μg of anti-6×His antibody (Merck Millipore, 05-949) for positive control and 4 μL of PBS buffer for negative control, respectively. Approximately 1 × 10^9 M13 phage particles were introduced into each reaction well. The complete reaction system was transferred to a 96-well PCR plate, sealed with optical sealing film, and incubated overnight at 4°C with constant rotation. Following incubation, 10 μL of Protein G Dynabeads (ThermoFisher, 10007D) were added to each well. After resealing, plates were incubated for an additional 4 hours at 4°C with rotation. Magnetic beads underwent six washing cycles with 200 μL of wash buffer containing 150 mmol/L NaCl, 50 mmol/L Tris-HCl, and 0.1% Tween 20 at pH 7.5, with each wash comprising eight complete pipette strokes. After the final wash, residual liquid was completely removed. Bound phage particles were eluted by resuspending beads in 30 μL of double-distilled water and heating at 95°C for 10 minutes.

**2.7.2. Sequencing Library Preparation**

Deep sequencing libraries were prepared through two rounds of PCR amplification using Hot Start Q5 Polymerase (New England Biolabs, M0493S) according to manufacturer specifications. Initial amplification employed forward primer S5-23R and reverse primer N7-18, both incorporating an 8-nucleotide barcode sequence (NNNNNNNN). The 50 μL reaction mixture contained 1× Q5 buffer, 200 μmol/L dNTPs, 0.5 μmol/L of each primer, and 0.5 μL polymerase. Thermal cycling parameters included: initial denaturation at 98°C for 2 minutes; 30 cycles of denaturation at 98°C for 10 seconds, annealing at 55°C for 30 seconds, and extension at 72°C for 30 seconds; final extension at 72°C for 2 minutes. Amplification products were purified by gel electrophoresis and quantified using NanoDrop 2000c spectrophotometry (Thermo Fisher Scientific). Secondary PCR utilized 25 ng of purified product as template with primers S510 and N710 in a 25 μL reaction system containing 1× Q5 buffer, 100 μmol/L dNTPs, 0.25 μmol/L primers, and 0.25 μL polymerase. Cycling conditions consisted of: 98°C for 2 minutes; 10 cycles of 98°C for 10 seconds, 55°C for 30 seconds, and 72°C for 30 seconds; final extension at 72°C for 2 minutes. Equimolar pools of amplification products from 96 samples were gel-purified, and the final library was sequenced using paired-end 150 bp reads on the Illumina HiSeq platform.

**2.7.3. Bioinformatic Processing and Peptide Quantification**

The library inserts, measuring 103 bp in length, were fully covered by paired-end sequencing. To ensure data integrity, only insert sequences demonstrating perfect concordance between paired-end reads with zero mismatches were retained for downstream analysis. Each insert sequence contained three distinct regions: an 8 bp N barcode, an 8 bp S barcode, and a 36 bp variable DNA region. The unique combination of N and S barcodes served as a sample-specific identifier, enabling precise assignment of each insert fragment to its corresponding serum sample. The 36 bp variable region was subsequently translated in silico into a 12-amino acid peptide sequence. Peptide sequences were mapped back to their respective serum samples using the dual barcode system, followed by comprehensive quantification of all peptide counts within each individual sample.

**2.7.4. Statistical Analysis and Functional Enrichment**

Raw data were processed with stringent quality control. Peptide counts were normalised and compared using the Wilcoxon rank-sum test. Significantly enriched peptides (p < 0.05, absolute fold change >2) were mapped to the human proteome via BLASTP against UniProtKB (e-value <1 × 10⁻⁵). For proteins matched by multiple peptides, the peptide with the highest |log₂(fold change)| was selected to represent differential reactivity. Enrichment analyses for Gene Ontology (GO), Kyoto Encyclopedia of Genes and Genomes (KEGG), and WikiPathway pathways were conducted using clusterProfiler (v4.12.1) with significance set at p < 0.05. This pipeline enabled unbiased identification of antibody signatures and related immune-pathophysiological pathways.
