# Supplementary material for: Waist‐to‐Body Mass Index Ratio and Risk of Musculoskeletal Disease in Type 2 Diabetes: A Cohort and PhIP‐Seq Analysis
Source: J Cachexia Sarcopenia Muscle. 2026 Aug 7;17(4):e70336. doi: 10.1002/jcsm.70336 (PMC13448775; doi:10.1002/jcsm.70336)
Supplement: Supplementary file 2 [file JCSM-17-e70336-s006.docx]

**DATA DICTIONARY**

**Study: Waist‑to‑Body Mass Index Ratio and Risk of Musculoskeletal Disease in T2DM**

This dataset contains anonymized clinical and laboratory data from a hospital-based cohort of adults with type 2 diabetes mellitus (T2DM). Each row represents one clinical assessment. Repeated measurements per participant are identifiable using **group_id_num**. Binary variables are coded as 1 = Yes / 0 = No unless otherwise specified.

**🔹 Identification & Follow‑up**

| **Variable** | **Full Name** | **Definition** | **Type** |
| --- | --- | --- | --- |
| date | Visit date | Date of clinical assessment (DD.MM.YYYY) | Date |
| group_id | Participant ID (original) | Unique patient identifier | String |
| group_id_num | Participant ID (numeric) | Numeric version of participant ID | Numeric |
| Group | Phenotype group | Cross-sectional phenotype classification (non-osteopenia/non-sarcopenia, osteopenia, sarcopenia, SOs) | Categorical |

**🔹 Demographics**

| **Variable** | **Full Name** | **Definition** | **Type** |
| --- | --- | --- | --- |
| Age_group | Age group | Age category (e.g., <65 / ≥65 years) | Binary |
| sex | Sex | 1 = Male, 2 = Female | Binary |
| age | Age | Age at assessment (years) | Continuous |
| duration_group | Diabetes duration group | ≤5 years / 5–15 years / >15 years | Categorical |
| Diabetes_duration | Duration of diabetes | Years since diagnosis of T2DM | Continuous |
| menopause | Menopausal status | 0 = Premenopausal, 1 = Postmenopausal | Binary |
| hysterectomy | History of hysterectomy | 0 = No, 1 = Yes | Binary |

**🔹 Anthropometric Measures**

| **Variable** | **Full Name** | **Definition** | **Unit** |
| --- | --- | --- | --- |
| BMI | Body mass index | Weight / height² | kg/m² |
| WC | Waist circumference | Measured at midpoint between rib and iliac crest | cm |
| WHtR | Waist-to-height ratio | WC / height | Ratio |
| WHR | Waist-to-hip ratio | WC / hip circumference | Ratio |
| WBR | Waist-to-BMI ratio | WC / BMI | Ratio |

**🔹 Laboratory Measurements**

| **Variable** | **Full Name** | **Unit** |
| --- | --- | --- |
| HGB | Hemoglobin | g/L |
| ALB | Albumin | g/L |
| ALT | Alanine aminotransferase | U/L |
| AST | Aspartate aminotransferase | U/L |
| eGFR | Estimated glomerular filtration rate | mL/min/1.73 m² |
| UACR | Urinary albumin-to-creatinine ratio | mg/g |
| TC | Total cholesterol | mmol/L |
| TG | Triglycerides | mmol/L |
| HDL | High-density lipoprotein cholesterol | mmol/L |
| LDL | Low-density lipoprotein cholesterol | mmol/L |
| HbA1c | Glycated hemoglobin | % |
| VID | 25-hydroxyvitamin D | ng/mL |
| CA | Serum calcium | mmol/L |

**🔹 Bone Parameters (DXA)**

| **Variable** | **Full Name** | **Unit** |
| --- | --- | --- |
| LumbarBMD | Lumbar spine bone mineral density (L1–L4) | g/cm² |
| LumbarT | Lumbar spine T-score | SD |
| LumbarZ | Lumbar spine Z-score | SD |
| FemurBMD | Femoral neck BMD | g/cm² |
| FemurT | Femoral neck T-score | SD |
| FemurZ | Femoral neck Z-score | SD |
| HipBMD | Total hip BMD | g/cm² |
| HipT | Total hip T-score | SD |
| HipZ | Total hip Z-score | SD |
| BodyBMD | Total body BMD | g/cm² |
| BodyT | Total body T-score | SD |
| BodyZ | Total body Z-score | SD |

**🔹 Muscle & Body Composition**

| **Variable** | **Full Name** | **Definition** |
| --- | --- | --- |
| ASMI | Appendicular skeletal muscle mass index | ASM / height² (kg/m²) |
| BF | Body fat percentage | Total fat mass / body weight (%) |
| BFobesity | Body fat–defined obesity | 1 = BF above sex-specific cutoff |
| Sarcopenia | Sarcopenia | Defined by ASMI criteria (1 = Yes) |
| Osteopenia | Osteopenia | T-score < –1.0 (1 = Yes) |
| SOb | Sarcopenic obesity | Sarcopenia + high BF (1 = Yes) |
| SOs | Sarcopenic osteopenia | Sarcopenia + osteopenia (1 = Yes) |

**🔹 Fracture Risk**

| **Variable** | **Full Name** | **Definition** |
| --- | --- | --- |
| Fracture | History or incident fracture | Radiologically confirmed |
| FRAX | 10-year probability of major osteoporotic fracture (MOF) | % |
| FRAX_hip | 10-year probability of hip fracture (HF) | % |

**🔹 Diabetic Complications**

| **Variable** | **Full Name** |
| --- | --- |
| DR | Diabetic retinopathy |
| DN | Diabetic nephropathy |
| DPN | Diabetic peripheral neuropathy |

(Binary: 1 = Yes, 0 = No)

**🔹 Comorbidities & Lifestyle**

| **Variable** | **Full Name** |
| --- | --- |
| Hypertension_history | History of hypertension |
| Hypertension | Hypertension (current diagnosis) |
| Smoking | Current smoking status |
| Drinking | Alcohol consumption |
| Exercise | Regular physical activity |

(Binary: 1 = Yes, 0 = No)

**🔹 Medications**

| **Variable** | **Full Name** |
| --- | --- |
| Biguanides | Biguanide therapy (e.g., metformin) |
| SGLT2 | SGLT2 inhibitor therapy |
| Other_GLDs | Other glucose-lowering drugs |
| Insulin_therapy | Insulin therapy |
| AHM | Antihypertensive medications |
| LLMD | Lipid-lowering medications |
| ZLLS | Zoledronic acid |

(Binary: 1 = Yes, 0 = No)
